# Supplementary material for: Evaluation of conditional treatment effect of salt stress on tomato sugar content using causal machine learning: A pilot study
Source: PLoS One. 2026 Jan 8;21(1):e0329424. doi: 10.1371/journal.pone.0329424 (PMC12782417; doi:10.1371/journal.pone.0329424)
Supplement: S1 Table — (DOCX) [file pone.0329424.s001.docx]

**S1 Table. Difference in average °Brix of tomato fruits between control and treatment.**

| Cultivation period | Mean  (Control) | Mean (Treatment) | Mean  Difference | p-value | Cohen's d | 95% CI (Lower) | 95% CI (Upper) |
| --- | --- | --- | --- | --- | --- | --- | --- |
| A | 6.0114 | 6.7600 | 0.7486 | < 0.0001 | 1.2326 | 1.8273 | 0.6270 |
| B | 5.6095 | 6.5762 | 0.9667 | < 0.0001 | 2.2931 | 2.8497 | 1.7275 |
| C | 5.6589 | 6.4600 | 0.8011 | < 0.0001 | 1.4003 | 1.8020 | 0.9930 |
| D | 5.6933 | 6.0167 | 0.3233 | 0.0772 | 0.6288 | 1.3157 | -0.0681 |
| E | 4.8349 | 5.3979 | 0.5630 | 0.0005 | 0.7521 | 1.1733 | 0.3269 |
| F | 6.2267 | 6.2917 | 0.0650 | 0.5295 | 0.1541 | 0.6321 | -0.3251 |
| G | 5.2382 | 6.1750 | 0.9368 | < 0.0001 | 1.3277 | 1.7471 | 0.9030 |
| H | 5.4972 | 7.5856 | 2.0883 | < 0.0001 | 1.9409 | 2.5926 | 1.2746 |
| I | 5.0278 | 6.2972 | 1.2694 | < 0.0001 | 2.0450 | 2.8538 | 1.2145 |

This table summarizes the mean, mean difference, p-value (by Welch's t-test), Cohen's d (standardized mean difference), and 95% confidence interval of the mean difference for each variable for the control and treatment groups at each cultivation period.
