# Supplementary material for: Evaluation of conditional treatment effect of salt stress on tomato sugar content using causal machine learning: A pilot study
Source: PLoS One. 2026 Jan 8;21(1):e0329424. doi: 10.1371/journal.pone.0329424 (PMC12782417; doi:10.1371/journal.pone.0329424)
Supplement: S2 Table — (DOCX) [file pone.0329424.s002.docx]

**S2 Table. Subgroup-Specific CATE derived from Causal Tree splits based on environmental conditions.**

| Subgroup | n (W=1 / W=0) | $\hat{y}$(T=1) | $\hat{y}$(T=0) | CATE | 95% CI |
| --- | --- | --- | --- | --- | --- |
| 1 | 50 / 40 | 0.3000 | 0.1000 | 0.2000 | [0.04, 0.36] |
| 2 | 44 / 42 | 0.8409 | 0.8095 | 0.0313 | [-0.14, 0.20] |
| 3 | 30 / 30 | 0.7000 | 0.1333 | 0.5666 | [0.35, 0.78] |
| 4 | 31 / 36 | 0.8064 | 0.1944 | 0.6120 | [0.41, 0.81] |
| 5 | 30 / 35 | 0.8666 | 0.2571 | 0.6095 | [0.41, 0.81] |
| 6 | 61 / 47 | 0.7704 | 0.0851 | 0.6853 | [0.55, 0.82] |
| 7 | 55 / 71 | 0.9454 | 0.1971 | 0.7482 | [0.63, 0.86] |

Each row represents a subgroup defined by a combination of covariate conditions identified by the Causal Tree. Treatment (W = 1) and control (W = 0) groups were compared within each subgroup. CATE represents the difference in outcome means between treated and control groups, with 95% confidence intervals (CI) calculated using standard error estimates.
