# Supplementary material for: Evaluation of conditional treatment effect of salt stress on tomato sugar content using causal machine learning: A pilot study
Source: PLoS One. 2026 Jan 8;21(1):e0329424. doi: 10.1371/journal.pone.0329424 (PMC12782417; doi:10.1371/journal.pone.0329424)
Supplement: Attachment — Submitted filename: PONE-D-25-38675_Response_to_reviewers_auresp_2.docx [file pone.0329424.s005.docx]

**Referees’ Comments & the Authors’ Responses**

**Comments from Referee #1:**

**Comment 1.** Brief explanation of Fig. 5 should be described in Result.

**Response 1.** Thank you for pointing this out. We agree with the reviewer’s suggestion. We have added a brief explanation of Fig. 5 in the Results section (page 21, line 10) to ensure that the figure is properly introduced and described.

**Comment 2.** Conclusion “Causal Tree analysis revealed that temperature and VPD jointly affected the treatment effect, with the strongest enhancement occurring under moderate conditions (20–25 °C, VPD < 0.84 kPa).” The condition 20–25 °C and VPD < 0.84 kPa caused minimum enhancement 0.031. For the strongest enhancement, the conditions was temperature < 20, VPD >= 0.84, and PAR < 13. Please revise the sentence. I apologize if I have misunderstood.

**Response 2.** Thank you very much for this insightful comment. After reviewing the figure again, we confirmed that the reviewer’s interpretation was correct and that our original description was inaccurate. We have therefore revised the relevant sentences in the Conclusion section (page 28, line 10-13). to accurately describe the conditions associated with the strongest enhancement. We appreciate the reviewer’s careful examination of our work.
